# Controlled Synthesis of PtNi Hexapods for Enhanced Oxygen Reduction Reaction

**DOI:** 10.3389/fchem.2018.00468

**Published:** 2018-10-04

**Authors:** Xing Song, Shuiping Luo, Xiaokun Fan, Min Tang, Xixia Zhao, Wen Chen, Qi Yang, Zewei Quan

**Affiliations:** Department of Chemistry, Southern University of Science and Technology (SUSTech), Shenzhen, China

**Keywords:** hexapod, platinum-nickel alloy, nanocrystal, oxidative etching, oxygen reduction reaction

## Abstract

Well-defined PtNi nanocrystals represent one of the most efficient electrocatalysts to boost the oxygen reduction reaction (ORR), especially in the shape of octahedrons, nanoframes, and nanowires. However, the synthesis of complex PtNi nanostructure is still a great challenge. Herein, we report a new class of PtNi hexapods with high activity and stability toward ORR. The hexapods are prepared by selective capping and simultaneous corrosion. By controlling the oxidative etching, PtNi polyhedrons and nanoparticles are obtained, respectively. The intriguing hexapods are composed of six nanopods with an average length of 12.5 nm. Due to their sharp tips and three-dimensional (3D) accessible surfaces, the PtNi hexapods show a high mass activity of 0.85 A mg${}_{\text{Pt}}^{-1}$ at 0.9 V vs. RHE, which are 5.4-fold higher than commercial Pt/C, also outperforming PtNi polyhedrons and PtNi nanoparticles. In addition, the mass activity of PtNi hexapods maintains 92.3% even after 10,000 potential cycles.

## Introduction

Proton exchange membrane fuel cells (PEMFCs) represent as one of the most promising clean energy technologies for electric vehicles, but the high cost of components and the sluggish kinetics of oxygen reduction reaction (ORR) hinder its broad deployment, especially when the expensive and scarce Platinum (Pt) is used as catalyst (Gasteiger et al., 2005). Thus, intensive efforts have been focused on improving the activity, stability and utilization of Pt catalyst (Huang et al., 2015; Zhang et al., 2015a; Wang et al., 2018). Specially, controlling the composition and morphology of Pt-based nanocrystals is demonstrated to be one of the most efficient strategies (Stamenkovic et al., 2006; Yin et al., 2012; Porter et al., 2013; Li and Sun, 2016). Marković and co-workers have reported that the bulk alloy of Pt_3_Ni composition with Pt (111)-skin exhibits greatly enhanced ORR activity compared with Pt. Pt_*x*_Ni alloy nanocrystals have been widely developed and which show the best catalytic performance toward ORR, outperforming the commercial Pt/C and other Pt-based catalysts with different component (Stamenkovic et al., 2007a,b; Choi et al., 2013). Therefore, engineering PtNi nanocrystals with well-defined shape, phase and composition is the subject of intensive interest to exploit their possible properties and applications (Cao et al., 2017; Zhang et al., 2018).

Apart from its composition, it is well known that the catalytic properties of Pt-based nanocrystals are closely related to their morphology, which determines the utilization efficiency and atomic arrangement of the active Pt atoms (Lim et al., 2009; Wu et al., 2012b; Quan et al., 2013; Li et al., 2014). Therefore, many research groups have synthesized Pt-based nanocrystals with various shapes, including polyhedrons (Chen et al., 2009), nanowires (Li et al., 2016; Jiang et al., 2017), nanodendrites (Huang et al., 2013), nanoplates (Bu et al., 2016), nanoframes (Chen et al., 2014; Luo et al., 2017) and so on. In particular, nanodendrites with porous structure or multi-branches possess high utilization of Pt atoms and high catalytic performance, benefiting from their three-dimensional (3D) accessible active sites and the tip-effect (Huang et al., 2013; Khan et al., 2016). Notably, multi-pod structure (tripod, tetrapod, pentapod, hexapod, octopod, etc.) with specific branches combines the advantages of nanodendrites and nanowires, possessing larger accessible surface areas (Teng and Yang, 2005; Zhang et al., 2010a; Lim and Xia, 2011). However, the controlled synthesis of well-defined PtNi multi-pod nanocrystals is still a great challenge.

Herein, we report the facile synthesis of well-defined PtNi hexapods through selective capping and oxidative etching. The as-prepared PtNi hexapods possess six pods with a length of 12.5 nm and 3D accessible surfaces. For comparison, PtNi polyhedrons and near-spherical nanoparticles are synthesized by controlling the oxidative etching. Electrochemical characterizations show that the PtNi hexapods exhibit enhanced catalytic activity toward ORR, compared with the PtNi polyhedrons, PtNi nanoparticles and commercial Pt/C. In addition, the PtNi hexapods show impressive stability, with negligible loss of activity after 10,000 cycles.

## Materials and methods

### Chemicals

Platinum(II) acetylacetonate [Pt(acac)_2_, 98%] and nickel(II) acetylacetonate [Ni(acac)_2_, 98%] were purchased from Energy Chemical. Oleylamine (OAm, 70%) was purchased from Sigma-Aldrich. Hexacarbonyl tungsten [W(CO)_6_, 97%] was purchased from Alfa Aesar. Ammonium bromide (NH_4_Br) was purchased from Aladdin. Solvents such as hexane, ethanol, methanol, and n-butylamine were analytical grade and used as received without further purification. The water used in all experiments was deionized (18.2 MΩ).

### Synthesis of PtNi hexapods

In a typical synthesis of PtNi hexapod nanocrystals, 0.05 mmol Pt(acac)_2_, 0.04 mmol Ni(acac)_2_ and 0.34 mmol NH_4_Br were dissolved in 8.0 mL OAm. The mixture was loaded into a three-neck flask equipped with a condenser and a thermometer under argon flow. When heated to 130°C under Ar atmosphere with gentle magnetic stirring, 0.14 mmol W(CO)_6_ powder was quickly added into the homogeneous solution. Afterwards, the reaction temperature was increased to 240°C at a rate of ~10°C/min and kept for 45 min. After the solution was cooled down to room temperature, the final products were precipitated by centrifugation, washed twice with ethanol and hexane, and further dispersed in hexane.

### Synthesis of PtNi polyhedrons

In a typical synthesis of PtNi polyhedrons, 0.05 mmol Pt(acac)_2_, 0.04 mmol Ni(acac)_2_, 0.34 mmol NH_4_Br, and 0.14 mmol W(CO)_6_ powder were dissolved in 8.0 mL OAm. After being degassed under vacuum, the mixture was heated to 240°C and kept for 45 min under argon flow. The final products were precipitated by centrifugation, washed twice with ethanol and hexane, and further dispersed in hexane.

### Synthesis of PtNi nanoparticles

The procedure was exactly the same as that for PtNi hexapods, except exposing the reaction system to air after adding 0.14 mmol W(CO)_6_ powder.

### Characterizations

X-ray diffraction (XRD) patterns were recorded on an X-ray diffractometer (Rigaku SmartLab) with Cu Kα radiation (λ = 0.15418 nm) at a voltage of 45 kV and a current of 200 mA. Transmission electron microscope (TEM) images were obtained by using FEI Tecnai F30 electron microscope (300 kV). High-angle annular dark-field scanning TEM (HAADF-STEM) and energy-dispersive X-ray (EDX) elemental mapping were obtained by using FEI Talos TEM. TEM samples were prepared by placing a drop of the nanoparticle colloidal solution onto a carbon-coated Cu grid under ambient conditions. The metal compositions and loading of catalysts were measured by inductively coupled plasma mass spectrometry (ICP-MS, Agilent 7700x).

### Electrochemical study

As for the electrochemical tests, the Pt loading of the PtNi nanocrystals loaded on carbon powders was controlled in the range of ~15%. The actual loadings of Pt were determined by ICP-MS. In a standard preparation, the PtNi nanocrystals were dispersed in 10 mL of hexane and Vulcant-72 carbon was dispersed in 15 mL of ethanol, then they were mixed and ultrasonicated for 30 min. Then, the carbon supported PtNi nanocrystals were collected by centrifugation and redispersed in 20 mL of n-butylamine. The mixtures were ultrasonicated for 30 min and then stirred at room temperature for 12 h. The products were collected by centrifugation and then washed with 20 mL methanol two times. The prepared catalysts were dried in vacuum at 80°C for 12 h. Finally, the pulverized samples were placed in a furnace preheated at 200°C and then heated for 1 h in air. The final catalyst suspension was prepared by mixing 4.0 mg of catalysts in 2.0 mL solution containing 1.8 mL ethanol and 0.2 mL 0.5 wt.% Nafion solution followed by ultrasonication for 30 min. Five Microliter catalyst suspension was dropped onto the clean Glassy carbon (GC) surface by using a micropipette and dried to form a uniform thin film at room temperature.

The electrochemical measurements were conducted in a three-electrode electrochemical cell with a Pine rotational disk electrode system connected with a biopotentiostat (AFCBP1E, Pine Instrument Co., USA) at 25°C. GC electrode (5 mm in diameter) covered with catalysts was served as a working electrode. A reversible hydrogen electrode (RHE) was used as a reference electrode and a Pt wire was used as a counter electrode. Cyclic voltamogram (CV) curves of catalysts in Ar-saturated 0.1 M HClO_4_ solution recorded at a scan rate of 50 mV s^−1^. The electrochemical surface areas (ECSAs) were measured by integrating the electric charges of hydrogen desorption region from 0.05 to 0.4 V on the CV curves carried out in Ar-saturated 0.1 M HClO_4_ solution. ORR measurements were conducted in O_2_-saturated 0.1 M HClO_4_ solution. The ORR polarization curves were collected at 1600 rpm with scan rate of 10 mV s^−1^. The durability tests were performed at 25°C in O_2_-saturated 0.1 M HClO_4_ solution by applying cyclic potential sweeps between 0.6 and 1.1 V vs. RHE at a scan rate of 100 mV s^−1^ for 10,000 cycles.

## Results and discussion

**Figure 1:**
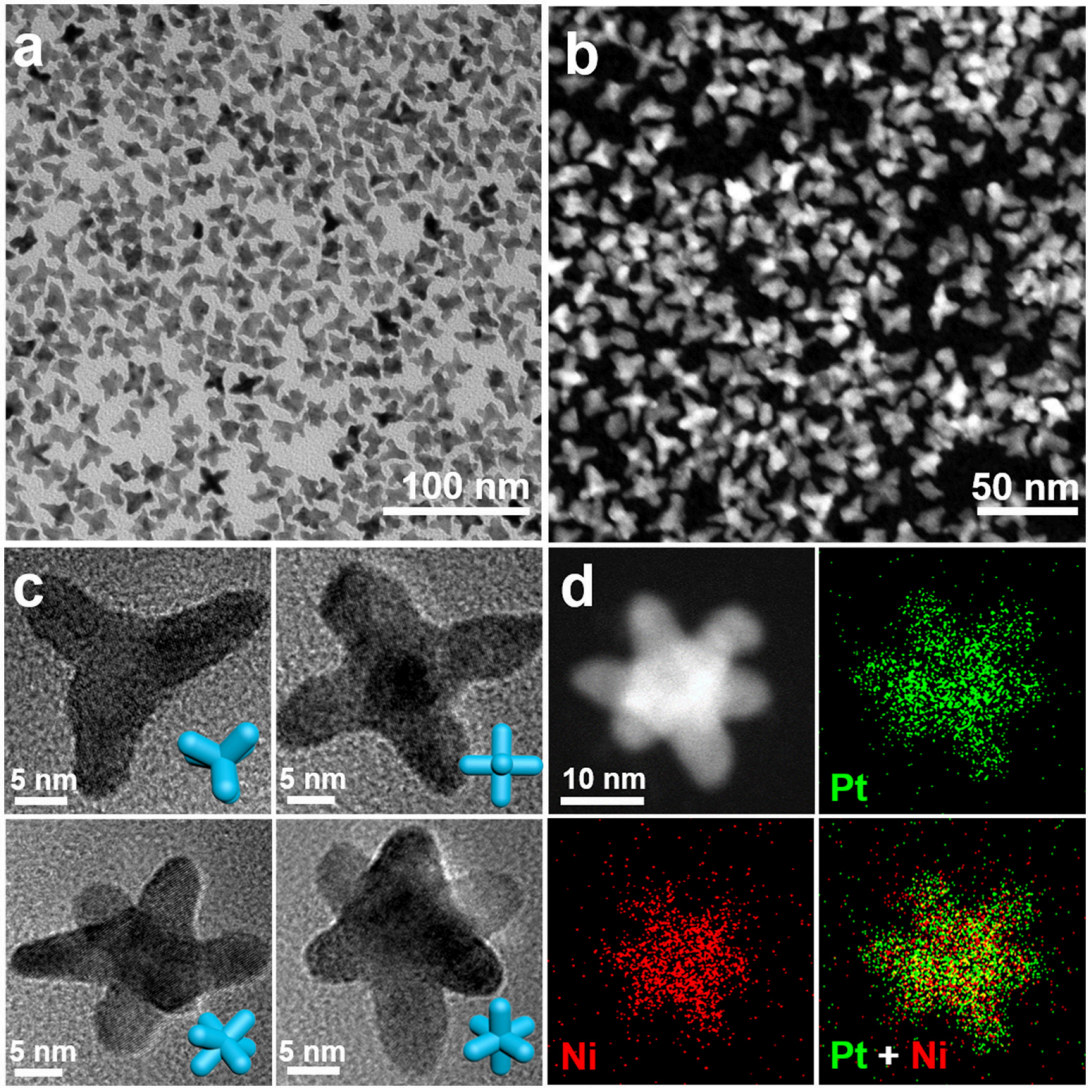
**(a)** Low-magnification TEM image and **(b)** HAADF-STEM image of PtNi hexapods. **(c)** The representative TEM images of nanocrystal obtained at different projection directions. **(d)** HAADF-STEM image and elemental mapping images of an individual PtNi hexapod. The insets of **(c)** show the corresponding 3D structure models of the nanocrystals.

Figures 1a,b show the TEM image and HAADF-STEM image of the as-obtained nanocrystals. The TEM image indicates that the well-dispersed nanocrystals are multi-branched nanostructure in high yield. The pod-like features in the nanocrystals can be clearly observed by TEM and STEM. Figure 1c shows the TEM images of typical PtNi nanostructures at different projections, including tripod, tetrapod, pentapod and hexapod, corresponding to the hexapod nanostructures oriented at different angles. To confirm the hexapod morphology in more details, we acquired a set of tilted TEM images at different α direction and illustrated their structure models. The TEM images of a typical hexapod tilted from 0° to 40° and their corresponding tilted 3D hexapod models shown in Figure S1. In particular, the projection of nanocrystal transforms from pentapod to hexapod through rotating a certain angle, which proves the hexapod structure of PtNi nanocrystals.

The high-resolution TEM (HRTEM) image of an individual PtNi hexapod oriented along the [001] zone axes (tetrapod) and its corresponding fast Fourier transform (FFT) pattern are shown in Figure S2, indicating the nanocrystals are highly crystallized. The lattice spacing (0.193 nm) in the pod region can be clearly observed, which demonstrates that the pod grows along the <100> directions. As shown in the XRD pattern of hexapod nanocrystals (Figure S3), the typical diffraction peaks locate between monometallic face-centered-cubic (*fcc*) Pt and Ni, demonstrating that the PtNi hexapods with a pure alloy phase are obtained. Figure 1d shows the STEM image and EDX elemental mapping images of an individual hexapod nanocrystal. The result indicates that Pt and Ni atoms are homogeneously distributed in this nanostructure. The overall Pt/Ni atomic ratio is determined to be 2.2/1.0 by the ICP-MS.

**Figure 2:**
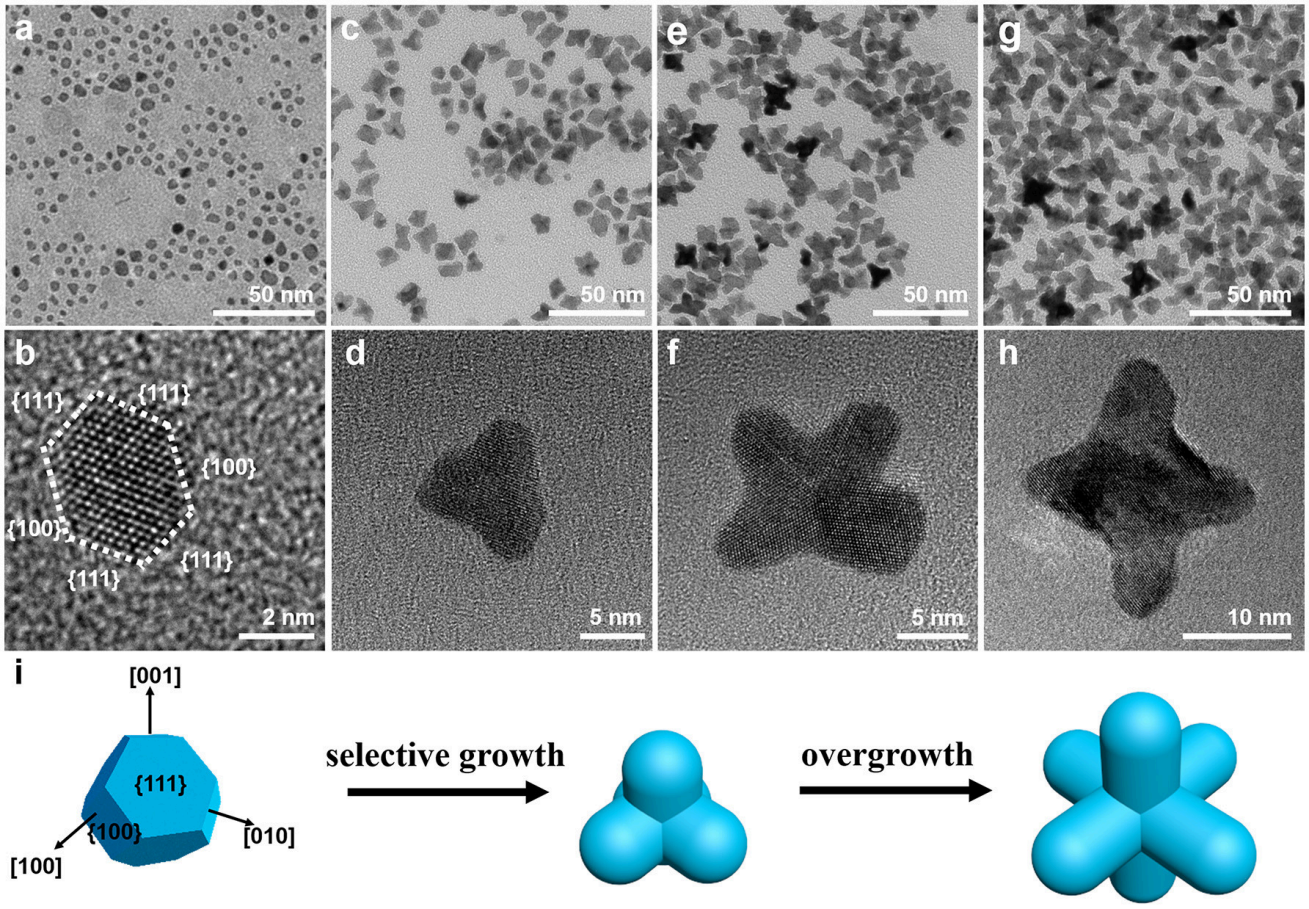
Representative TEM and HRTEM images of PtNi nanocrystals obtained at different reaction stages: **(a,b)** 140°C, **(c,d)** 150°C, **(e,f)** 160°C, **(g,h)** 200°C. **(i)** Schematic of growth mechanism illustrating the morphological evolution of the PtNi hexapods.

To understand the growth mechanism of PtNi hexapods, the intermediate products obtained at different reaction stages were characterized by TEM (Figure 2). During the heat-up synthesis, the solution turned brown at 140°C, and the products were separated immediately. As shown in the TEM image (Figure 2a), small nanoparticles with a diameter of 5.0 nm were formed at this stage. It is clearly observed in the HRTEM image (Figure 2b) that the nanoparticle possesses the shape of truncated octahedron, which is surrounded by {100} and {111} facets. As known, due to the low surface energy, polyhedron consisting of mixed {100} and {111} facets is a typical thermodynamically stable shape at the nucleation stage (Wu et al., 2012a; Gan et al., 2014). When the temperature was further raised to 150°C (Figures 2c,d), the pods with a length of ~5.0 nm can be observed, which is much shorter than that for the final product. The six pods are formed by the selective growth along the <100> directions on truncated octahedral seeds (Gan et al., 2014). At 160°C, most nanoparticles transform into multi-branched nanostructure through rapid growth along the <100> directions, and the length of pods increases to ~8.0 nm, as shown in Figures 2e,f. By further increasing the temperature to 200°C, the length of pods reached to ~12.0 nm, indicating the continuous growth of hexapod with increasing temperature (Figures 2g,h). It is clear to illustrate the growth trajectory of PtNi hexapods as schematically represented in Figure 2i. Based on above observations, the growth of PtNi hexapod nanocrystals follows the classic nucleation-growth mechanism, including the formation of stable polyhedral seeds and the selective growth along the <100> directions.

During this synthesis, NH_4_Br and W(CO)_6_ play key roles in the formation of PtNi hexapods. It has been reported that Br ions (Br^−^) can selectively adsorb on {100} surfaces (Li et al., 2015; Luo and Shen, 2017), and carbon monoxide (CO) produced by the decomposition of W(CO)_6_ can selectively adsorb on the {111} facets of PtNi nanocrystals (Zhang et al., 2010b). Thus, the truncated octahedron consisting of mixed {100} and {111} facets are expected to produce. In addition, the Br^−^/O_2_ pairs are the most common reagent for etching transition metals, such as Ni (Wiley et al., 2004; Chen et al., 2012). The Br^−^ capped on the {100} facets in presence of O_2_ would cause the etching of {100} facets and exposing of specific active sites. As a result, the unstable Ni atoms can be partially dissolved by the Br^−^/O_2_ pairs, leading to the activated {100} facets. Then, the newly formed atoms prefer to deposit on these sites, and keep growing along the <100> directions (Wang et al., 2016; Tang et al., 2018). At the same time, the adsorption of CO on the {111} facets is stronger than the adsorption of Br^−^ on the {100} facets, hindering the growth along <111> directions (Huang et al., 2010). Thus, we propose that the formation of PtNi hexapod nanocrystals is ascribed to the selective capping and oxidative etching (Luo and Shen, 2017).

**Figure 3:**
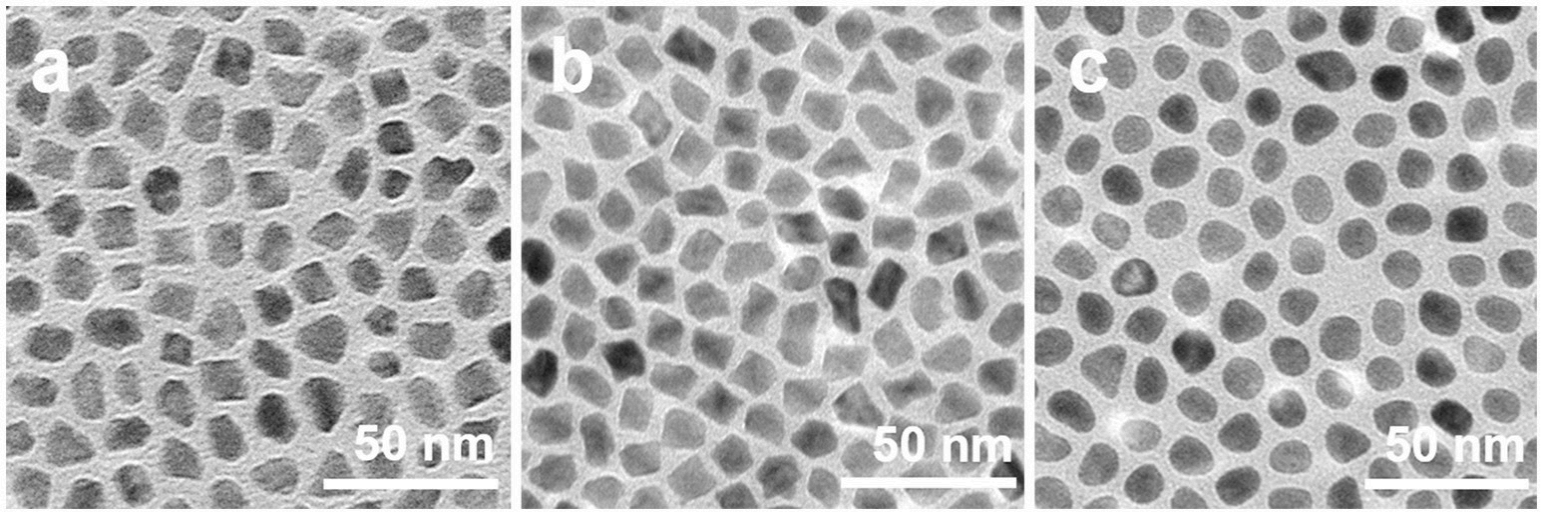
TEM images of PtNi nanocrystals obtained by using the standard procedures **(a)** in the absence of NH_4_Br, **(b)** in the absence of O_2_, and **(c)** with the system exposed to air.

To confirm above selective capping and oxidative etching formation mechanism of PtNi hexapods, we designed a set of control experiments. As shown in Figure S4, PtNi nanocrystals can be produced in the absence of W(CO)_6_, but their morphology is irregular with larger size. Such result indicates that the presence of W(CO)_6_ is essential for the size and shape control of the PtNi hexapod nanocrystals. In the absence of NH_4_Br, the PtNi polyhedral nanocrystals are obtained (Figure 3a), indicating that the NH_4_Br is indispensable to form the pod-like nanostructure. Without NH_4_Br, the capping and etching effects of Br^−^ are eliminated, therefore, the morphology of polyhedron is regulated by the W(CO)_6_. On the other hand, during the synthesis of PtNi hexapod nanocrystals, O_2_ could be introduced into the solution along with the addition of W(CO)_6_. As a comparison, we completely removed O_2_ in the system by degassing the sealed three-necked flask for 30 min at room temperature. As shown in Figure 3b, PtNi polyhedron nanocrystals are obtained. The oxidative etching effect on the nanocrystals disappears when O_2_ is absent. This result indicates that the oxidative etching is necessary to the formation of hexapod nanocrystals by inducing the selective growth along <100> directions. In addition, when the system is exposed to air (Figure 3c), PtNi nanoparticles with near-spherical shapes without sharp tips and extended surfaces are obtained, due to the stronger oxidative etching ability of Br^−^/O_2_ in the presence of excessive O_2_. Thus, the successful formation of PtNi hexapods is attributed to the selective capping and moderate oxidative etching.

## Electrochemical properties

**Figure 4:**
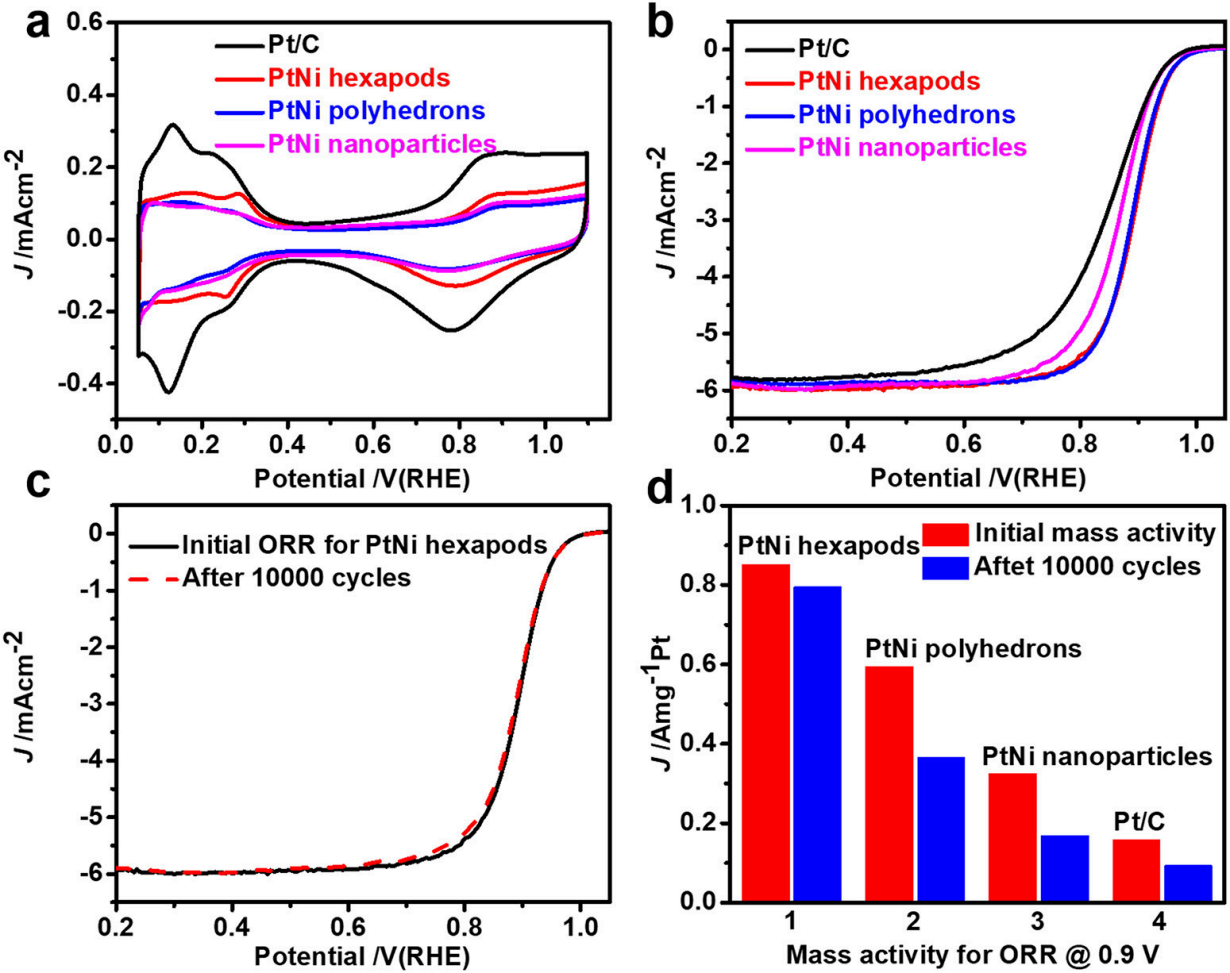
**(a)** CV curves of PtNi hexapods, polyhedrons, nanoparticles and commercial JM Pt/C catalyst recorded at a scan rate of 50 mV s^−1^ in Ar-saturated 0.1 M HClO_4_ solution; **(b)** ORR polarization curve of these catalysts at a scan rate of 10 mV s^−1^ in O_2_-saturated 0.1 M HClO_4_ solution; **(c)** ORR polarization curve of PtNi hexapods before and after 10,000 potential cycles; **(d)** Mass activity of different catalysts before and after 10,000 potential cycles.

The electrochemical properties of the as-prepared PtNi hexapods, PtNi polyhedrons, PtNi nanoparticles and commercial Pt/C from the Johnson Matthey (20% Pt, JM) were investigated and compared under the same conditions. Before the electrochemical measurements, the products were treated with n-butylamine to exchange shorter ligands around the nanocrystals (Cargnello et al., 2015) and heated in air at 200°C for 1 h. Finally, the carbon supported products were dissolved in the mixed nafion and ethanol solution by ultrasonication. Figure 4a shows CV curves of the PtNi hexapods, PtNi polyhedrons, PtNi nanoparticles and commercial Pt/C in Ar-saturated 0.1 M HClO_4_ solution at a scan rate of 50 mV s^−1^. The electrochemical surface areas (ECSAs), measured by integrating the electric charges of hydrogen desorption region from 0.05 to 0.4 V, are 47.2 m^2^ g^−1^ for PtNi hexapods, 23.9 m^2^ g^−1^ for PtNi polyhedrons and 21.4 m^2^ g^−1^ for PtNi nanoparticles, respectively. Although the size of PtNi hexapods is far larger than the commercial Pt/C, the ECSA is approximate to it (48.4 m^2^ g^−1^), which is due to the presence of sharp tips in these PtNi hexapods.

The ORR polarization curves of different electrocatalysts (Figure 4b) were performed at a scan rate of 10 mV s^−1^ in O_2_-saturated 0.1 M HClO_4_ solution via rotating disk electrode approach. The half-wave potential of PtNi hexapods is positively shifted by 49 mV than that of commercial Pt/C. Additionally, the PtNi hexapods exhibit the greatest mass activity of 0.85 A mg${}_{\text{Pt}}^{-1}$ at 0.9 V vs. RHE, which is 5.4 times higher than that of commercial Pt/C (0.16 A mg${}_{\text{Pt}}^{-1}$), which is attributed to the synergistic effect of Pt-Ni alloy (Zhang et al., 2015b) and 3D accessible surfaces. Furthermore, the mass activity of PtNi hexapods is 1.4 and 2.6 times higher than PtNi polyhedrons (0.59 A mg${}_{\text{Pt}}^{-1}$) and PtNi nanoparticles (0.32 A mg${}_{\text{Pt}}^{-1}$), respectively.

The electrochemical durability tests of these three PtNi catalysts and commercial Pt/C were recorded by sweeping from 0.6 to 1.1 V vs. RHE for 10,000 cycles at a scan rate of 100 mV s^−1^. As shown in Figure 4c, the half-wave potential of PtNi hexapods is negligibly degraded. Figure 4d indicates that the mass activity for PtNi hexapods after durability tests remains 92.3% of its initial value. However, under the same conditions, the mass activity of PtNi polyhedrons, PtNi nanoparticles and commercial Pt/C only remain 61.5, 52.0, and 58.9%, respectively, and exhibit a large negative shifting compared with the initial ORR polarization curves (Figure 4d, Figure S5). The TEM images reveal the structures of catalysts before and after the durability tests (Figure S6), which indicates that PtNi hexapods have negligible change on the morphology. After detailed EDX analysis (Figure S7), the average molar ratio of Pt/Ni before and after durability test are determined to be 2.12/1.0 and 2.26/1.0, respectively. The results demonstrate that the molar ratios of Pt and Ni are well maintained after 10,000 cycles, further confirming the stability of PtNi hexapods. However, under the same conditions, the PtNi polyhedrons and PtNi nanoparticles exhibit obvious changes on the morphology, while the Pt/C catalysts are changed to larger particles and seriously aggregated after 10,000 cycles. The results indicate that these PtNi hexapods have an impressive electrochemistry stability compared with the commercial Pt/C.

## Conclusion

In summary, we have successfully synthesized the well-defined PtNi hexapod nanocrystals with 3D accessible surfaces. The formation of truncated octahedron seeds, selective growth and moderate oxidative etching synergistically govern the synthesis of PtNi hexapods. Notably, the PtNi hexapods exhibit much enhanced electrocatalytic activity for ORR. In addition, the PtNi hexapods retain high activity and stability after 10,000 cycles. Our work provides a facile route to engineer multi-pod nanostructure with high catalytic performance, which advances the understanding of the relationship between nanoparticle geometry and catalytic performance.

## Author contributions

ZQ conceived the project. XS carried out the experiments and collected the data. SL performed the electrochemical tests. XF, MT, XZ, WC, and QY made contribution to the analysis and discussion of the results. XS drafted the manuscript, and all authors contributed to the final version of the manuscript.

### Conflict of interest statement

The authors declare that the research was conducted in the absence of any commercial or financial relationships that could be construed as a potential conflict of interest.
