# Supplementary material for: Controlled Synthesis of PtNi Hexapods for Enhanced Oxygen Reduction Reaction
Source: Front Chem. 2018 Oct 4;6:468. doi: 10.3389/fchem.2018.00468 (PMC6180145; doi:10.3389/fchem.2018.00468)
Supplement: Supplementary file 1 [file Table_1.DOCX]

Supplementary Material

Controlled Synthesis of PtNi Hexapods for Enhanced Oxygen Reduction Reaction

Xing Song^1^, Shuiping Luo^1^, Xiaokun Fan^1^, Min Tang^1^, Xixia Zhao^1^, Wen Chen^1^, Qi Yang^1^, Zewei Quan^1^*

^1^Department of Chemistry, Southern University of Science and Technology (SUSTech), Shenzhen, Guangdong 518055, P. R. China.

*** Correspondence:**Zewei Quan
quanzw@sustc.edu.cn


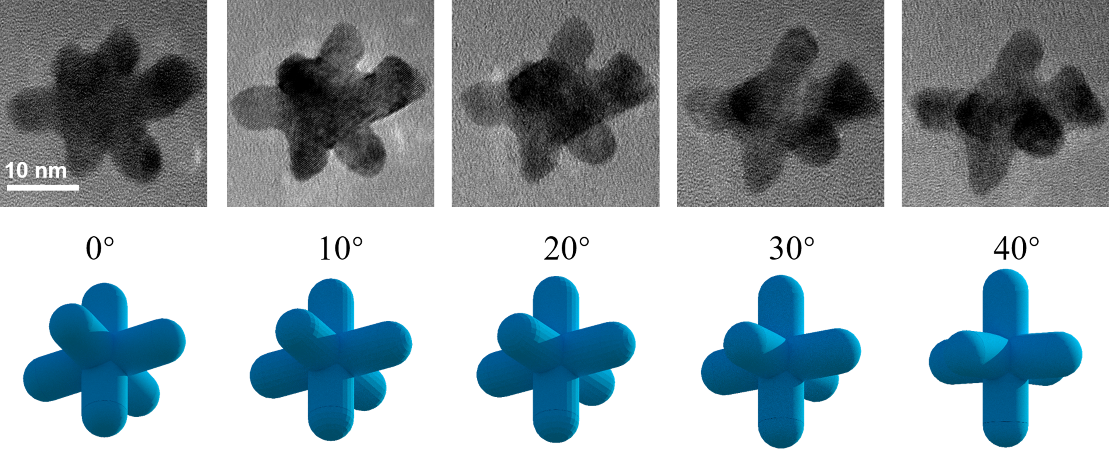


**Figure S1.** A series of TEM images of an individual hexapod obtained at different tilting degrees (0°, 10°, 20°, 30°, and 40°), and their corresponding hexapod structural modes.

**
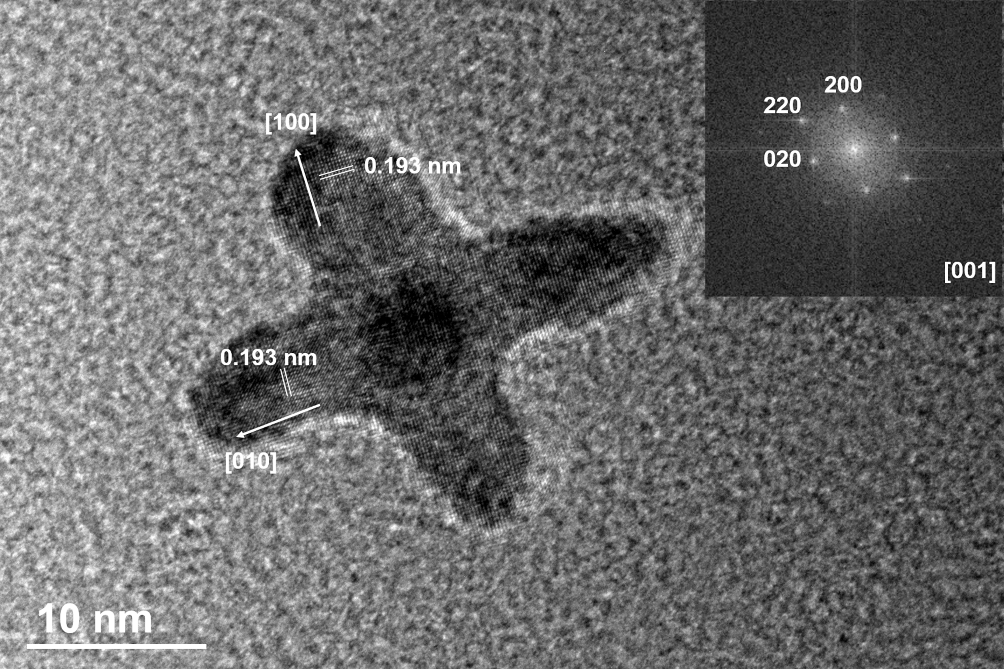
**

**Figure S2.** HRTEM image of an individual PtNi hexapod and the corresponding FFT pattern along the [001] zone axis.

**
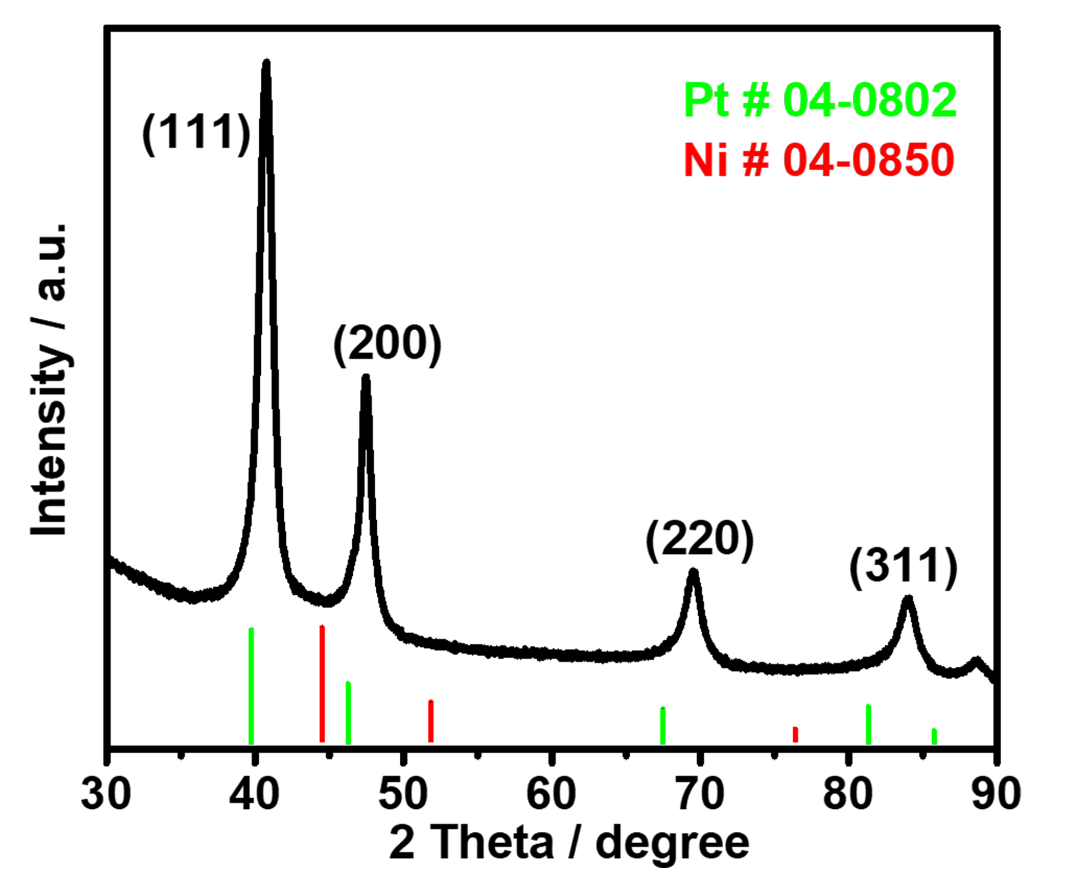
**

## Figure S3. XRD pattern of PtNi hexapod nanocrystals.


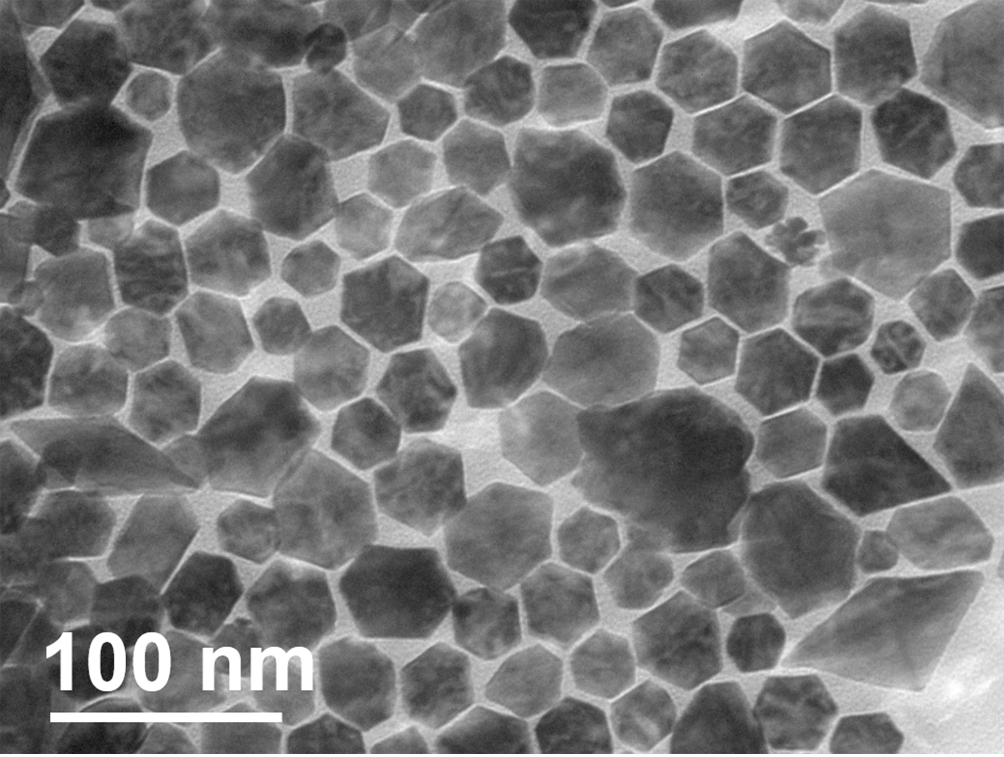


**Figure S4.** TEM image of PtNi nanocrystals obtained in the absence of W(CO)_6_.


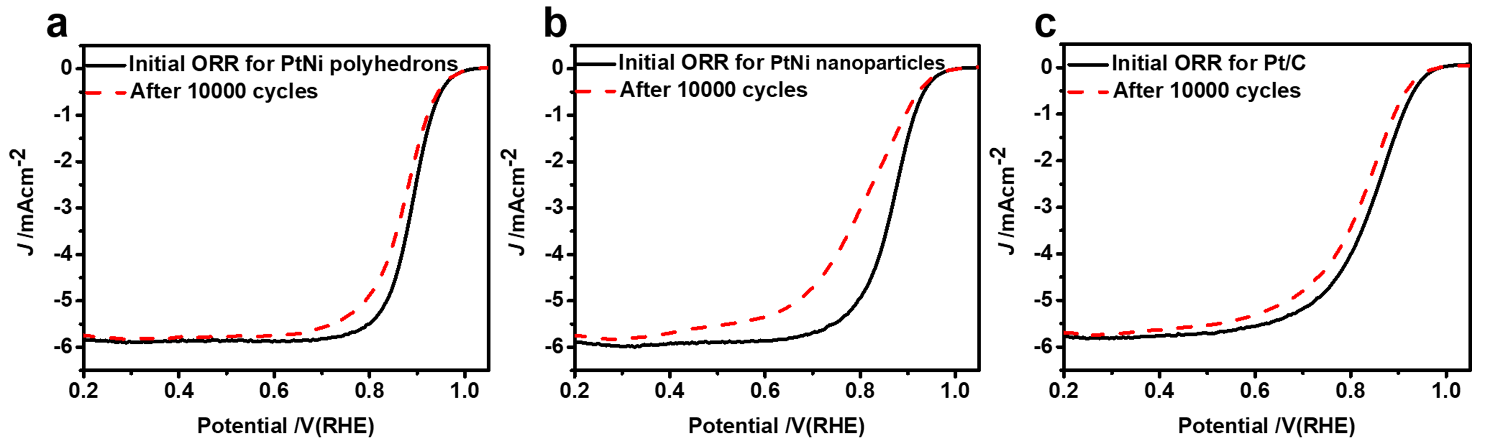


**Figure S5.** ORR polarization curves of (a) PtNi polyhedrons/C, (b) PtNi nanoparticles/C and (c) JM-Pt/C. electrocatalysts recorded before and after 10,000 cyclic voltammogram cycles between 0.6 and 1.1 V.


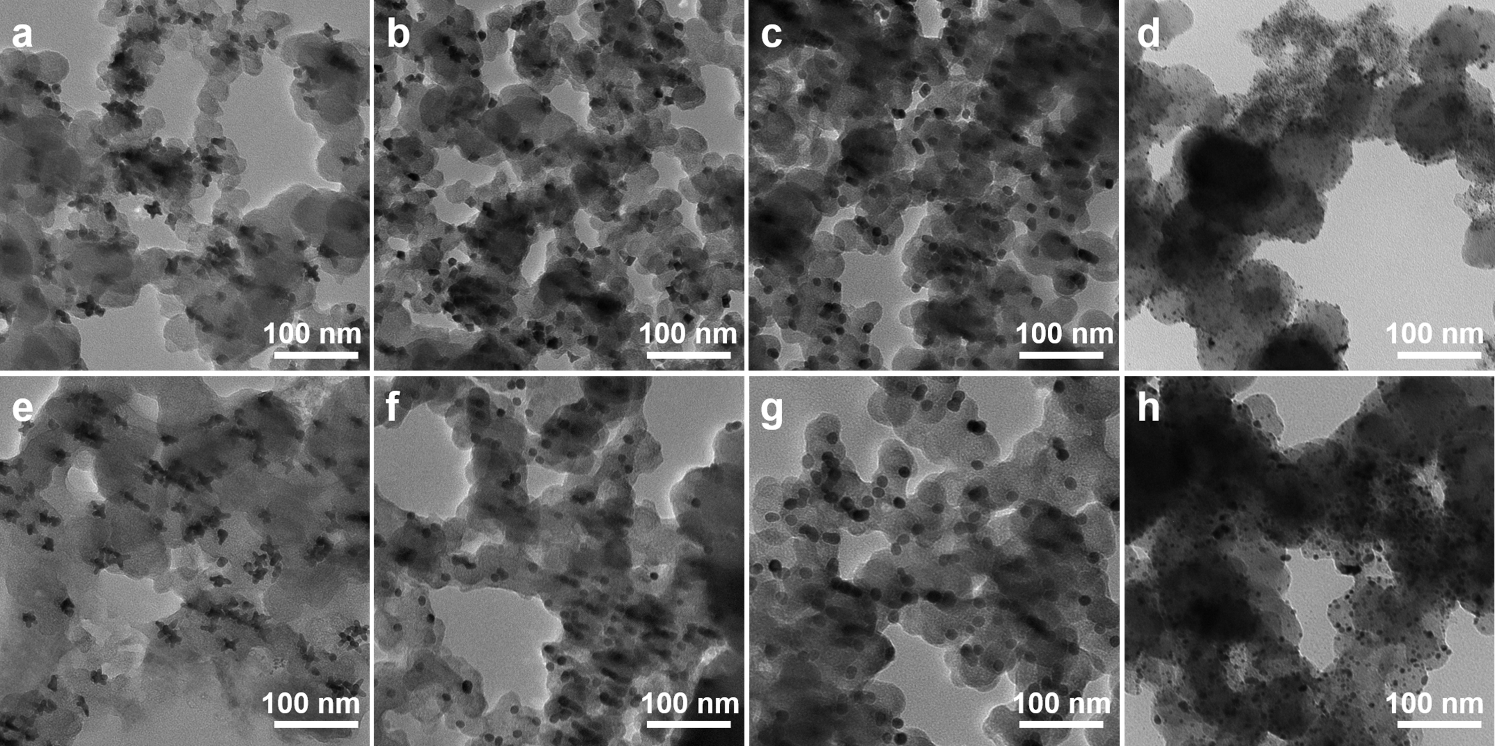


**Figure S6.** Representative (a-d) TEM images of the carbon supported PtNi hexapods, PtNi polyhedrons, PtNi nanoparticles and JM-Pt/C, respectively. (e-h) TEM images of the carbon supported PtNi hexapods, PtNi polyhedrons, PtNi nanoparticles and JM-Pt/C collected after 10000 cyclic voltammogram cycles between 0.6 and 1.1 V.


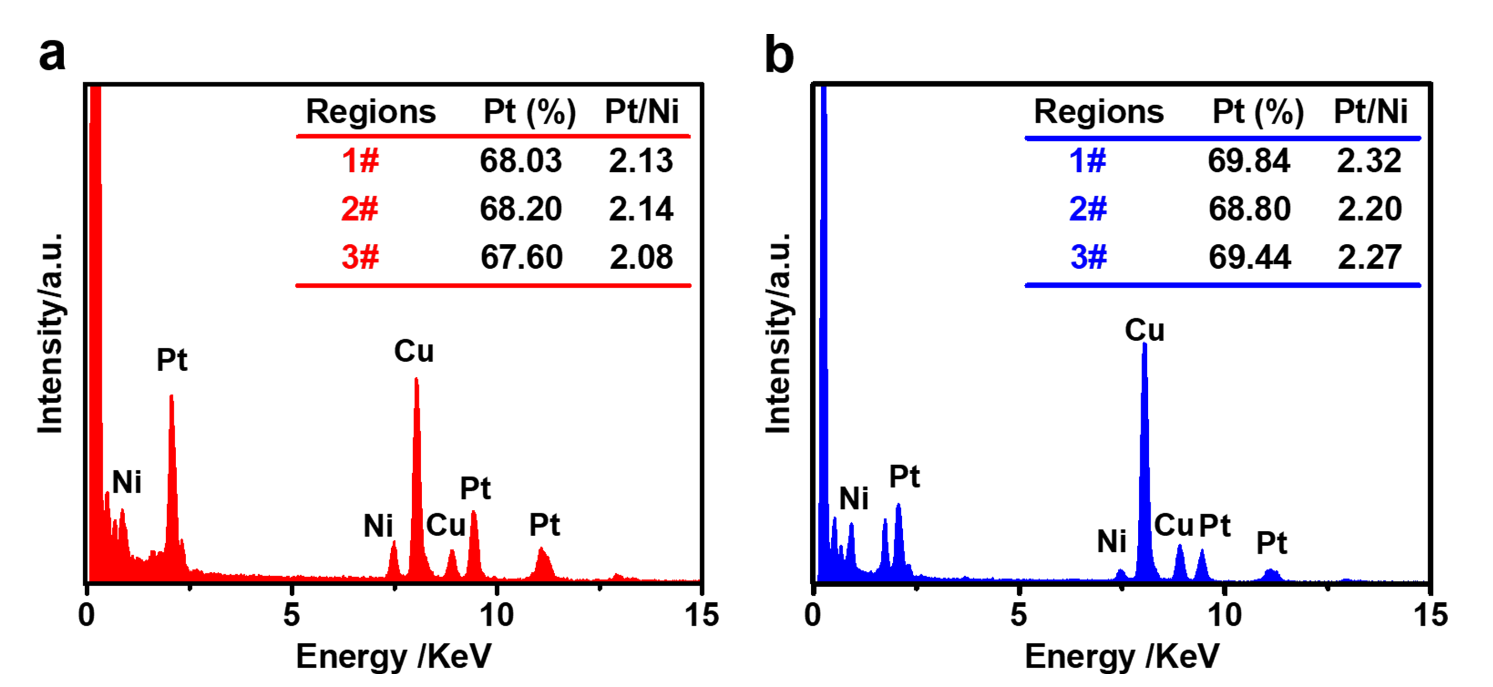


**Figure S7.** EDX spectra of the PtNi hexapods (a) before and (b) after 10000 potential cycles, respectively. The insets show the detailed composition analysis on three different regions from TEM grids.
